# Case Series: Re-induction of Intravenous, Weight-Based Ustekinumab Is Well Tolerated in Patients With Moderate–Severe Crohn’s Disease

**DOI:** 10.1093/crocol/otac012

**Published:** 2022-04-27

**Authors:** Scott David Lee, Kendra Kamp, Kindra Dawn Clark-Snustad

**Affiliations:** University of Washington, Division of Gastroenterology, Seattle, Washington, USA; University of Washington, Division of Gastroenterology, Seattle, Washington, USA; University of Washington, Division of Gastroenterology, Seattle, Washington, USA

**Keywords:** Crohn’s disease, ustekinumab, re-induction

## Abstract

**Background:**

Crohn’s disease (CD) patients may benefit from biologic optimization.

**Methods:**

We retrospectively assessed adverse events (AEs) and clinical/endoscopic response after ustekinumab re-induction in CD patients.

**Results:**

We identified 28 patients, all with prior biologic exposure. Eight weeks following re-induction, 10.7% reported ≥1 AE. Three serious AEs occurred in a single patient (CD flares). No infusion reactions occurred. 53.8% and 38.5% achieved clinical response and remission, respectively. 42.8% achieved both endoscopic improvement and remission.

**Conclusions:**

Ustekinumab re-induction was well tolerated. Clinical and endoscopic disease activity improved in some patients. Further larger studies are needed to verify these findings in a broader population.

## Introduction

While biologics are highly effective for Crohn’s disease (CD), only 30% of patients achieve endoscopic remission on standard dosing, and loss of response is common.^1,2^ Active CD increases the risk of strictures, abscesses, and fistulas which may require surgery and can cause short gut syndrome, malnutrition, and chronic symptoms. Achieving endoscopic remission reduces the risk of relapse compared to those with mild endoscopic disease.^3^ Studies suggest off-label dose optimization of biologics improves outcomes in some patients with CD.^4,5^ Additionally, patients may have interruption of biologic therapy due to poor adherence, insurance coverage issues, or medical indications (eg, severe infection, surgery) and may benefit from re-induction upon restarting biologic therapy.

Ustekinumab [Stelara®, Janssen] is a monoclonal antibody to the p40 subunit of interleukin-12 and interleukin-23 that is Food and Drug Administration (FDA) approved for CD with a weight-based intravenous induction followed by 90 mg subcutaneously every 8 weeks.^2^ Ustekinumab trials report clinical remission rates around 50%–65% after 44–52 weeks,^2,6^ suggesting that up to one-half of patients may not achieve remission of symptoms on standard dosing. Additionally, studies report an exposure–response relationship suggesting higher doses of ustekinumab, shortened intervals, or re-induction may improve endoscopic endpoints.^7^ Yet, data are limited on the safety and efficacy of these approaches.^8^ Studies have primarily focused on modification of the ustekinumab maintenance dose with a recent nationwide Finish study reporting similar responses in C-reactive protein (CRP) and fecal calprotectin between groups with and without dose modification.^9^ However, the limited number of objective endoscopic evaluations limits interpretation of these data. Retrospective studies report improved outcomes in patients receiving modification of the maintenance dose of ustekinumab.^8,10^ Small retrospective studies also report on the safety or effectiveness of re-induction with ustekinumab.^11–15^

With regard to safety, ustekinumab is well tolerated. Ustekinumab and other biologic therapies have not demonstrated dose-related side-effect profiles.^2,16–19^ However, the safety of re-induction with ustekinumab has not been specifically evaluated in clinical trials. Therefore, the aim of this study is to report on the safety and effectiveness of ustekinumab re-induction in patients with CD.

## Materials and Methods

We received institutional review board approval for this retrospective study. Eligible patients received care at a tertiary referral center or county hospital in Washington state, were ≥18 years old, received ≥1 ustekinumab re-induction infusion for CD between 2016 and 2021, and had ≥1 encounter following re-induction. This cohort includes some patients who received ustekinumab via UNITI-1, UNITI-2, or IM-UNITI clinical trials, then transitioned to clinical practice upon trial completion.^2^

### Data Collection

Age, body mass index, sex, tobacco use, age at diagnosis, disease duration/extent/behavior, gastrointestinal surgical history, prior CD therapy, and concomitant immunomodulator and corticosteroid use were collected retrospectively from medical records, or from UNITI-1, UNITI-2, and IM-UNITI sponsor data.

Harvey Bradshaw Index (HBI), Short Inflammatory Bowel Disease Questionnaire (SIBDQ), corticosteroid use, CRP, hematocrit, and albumin were collected prior to and after re-induction. Patients are asked to complete HBI and SIBDQ questionnaires at each clinical encounter per standard clinical practice. Simple Endoscopic Score in CD (SESCD) was collected prior to and after re-induction. SESCD is scored at the time of colonoscopy per standard practice.

### Definitions

#### Safety

Adverse events (AEs) were defined as any untoward medical occurrence during the study period, and were collected during therapy until 8 weeks after discontinuation or date of last data capture. Serious AEs (SAE) were defined as AEs that resulted in death, were life threatening, required inpatient hospitalization, prolonged hospitalization, or resulted in significant disability. AE causality was assessed by investigators as not related, unlikely related, possibly related, probably related, and highly probably related to ustekinumab.

#### Effectiveness

Effectiveness was predefined as: active clinical disease (baseline HBI ≥5); clinical response (≥3-point decrease in HBI); clinical remission (HBI ≤4); active endoscopic disease (baseline SESCD ≥6, or ≥4 for isolated ileal disease); endoscopic improvement (≥50% decrease in SESCD); endoscopic remission (SESCD ≤3); and endoscopic healing (SESCD = 0).

### Approach to Therapy

In our clinical practice, patients received ustekinumab re-induction for several reasons: to improve disease activity in patients with response, but not remission to standard dosing of ustekinumab, to regain response in patients with loss of response,^12^ to restart ustekinumab after a break in therapy, or to transition to standard clinical therapy after completing a clinical trial. Patients received re-induction with intravenous infusion of either ustekinumab 260, 390, or 520 mg according to weight per prescribing guidelines.^20^

### Statistics

Statistical analyses were completed in Stata (Stata Statistical Software: Release 17, StataCorp, College Station, TX, 2019). For continuous variables, summary statistics including mean, standard deviations, median, interquartile range (IQR), and ranges were calculated. For categorical variables including AEs and SAEs, we calculated proportions. To determine the effectiveness of ustekinumab re-induction, we report the proportion of patients meeting clinical, endoscopic, and laboratory endpoints. For clinical and endoscopic endpoints, paired *t*-tests were used to compare prior to and post re-induction values for patients with disease activity. Surgically absent bowel was scored as zero for endoscopic endpoints. Scores were excluded from analysis if missing, if surgical anatomy invalidated the score (eg, HBI), or if the patient did not meet the predefined score for active clinical or endoscopic disease at baseline.

## Results

Five hundred and sixteen patients with an administrative code for ustekinumab were identified, 253 were confirmed to have CD, 232 received ustekinumab for CD, and 28 received ustekinumab re-induction. Reasons for re-induction were: active clinical symptoms and active endoscopic disease (*n* = 7), active clinical symptoms only (*n* = 5), active endoscopic disease only (*n* = 3), active endoscopic disease on capsule (*n* = 1), routine transition to care following clinical trial (*n* = 6), and restarting ustekinumab following interruption in therapy (*n* = 6).

Twenty-six patients received a single re-induction dose. One patient remained on every 12-week infusions as maintenance therapy due to insurance denial of subcutaneous ustekinumab. One patient with secondary loss of response to ustekinumab received multiple re-induction doses over several years given response, then loss of response to each re-induction dose. For the 2 later patients, response to the first re-induction dose was utilized for analysis in this study.

### Ustekinumab Dosing and Duration

Mean ustekinumab treatment duration prior to re-induction was 28.7 months (SD 20.3). The ustekinumab maintenance dose prior to re-induction was 90 mg SC every 12 weeks (*n* = 3), every 8 weeks (*n* = 13), every 6 weeks (*n* = 1), and every 4 weeks (*n* = 1). Ten patients had a break in therapy prior to re-induction for a mean 19.5 months (SD 20.2); maintenance dosing prior to break in therapy was 90 mg every 8 weeks (*n* = 7) and no maintenance dosing (*n* = 3). Mean treatment duration after re-induction was 29.4 months (SD 16.9). At the end of data collection, 22 (78.6%) patients remained on ustekinumab; reason for discontinuation was secondary loss of response (*n* = 3), inadequate response (*n* = 2), and lost to follow-up (*n* = 1).

### Demographics and Disease Characteristics

The mean age was 40.0 years (SD 12.5), 57.1% were female, and median disease duration was 11 years (IQR 7.5–19.0). For disease extent, 14.3% had ileal, 32.1% had colonic, and 53.6% had ileocolonic disease. Half of patients had prior gastrointestinal surgery and all had prior exposure to ≥1 biologic. At the time of re-induction, 46.4% received concomitant thiopurine or methotrexate and 28.6% received concomitant corticosteroids (Table 1).

**Table 1.** Demographics and disease characteristics at time of ustekinumab re-induction for all patients with Crohn’s disease who received ustekinumab re-induction and for patients who received ustekinumab re-induction and contributed data to the clinical and/or endoscopic outcomes.

Characteristic	Patients contributing to safety analysis,*N* = 28	Patients contributing to clinical and/or endoscopic outcomes, *N* = 15
Mean age, years (SD)	40.0 (12.5)	41.5 (11.3)
Mean BMI,kg/m^kg m^−2^^ (SD)	25.1 (6.9)	26.7 (8.4)
Mean albumin, g/dL^−1^, reference range: 3.5–5.2 g dL^−1^, *n* (SD)	4.0 (0.6)	3.8 (0.6)
Female sex, *n* (%)	16 (57.1)	8 (53.3)
Tobacco use, *n* (%)		
Never	22 (78.6)	11 (73.3)
Current	2 (7.1)	1 (6.7)
Former	4 (14.3)	3 (20.0)
Age at diagnosis, *n* (%)		
Less than 17 years	7 (25.0)	2 (13.3)
17–40 years	19 (67.9)	11 (73.3)
Over 40 years	2 (7.1)	2 (13.3)
Median disease duration, years (IQR)	11 (7.5–19.0)	9 (7.0–18.0)
Mean duration of follow-up on standard dosing, years (SD)	2.4 (1.7)	1.8 (1.6)
Mean duration of follow-up after re-induction, years (SD)	2.5 (1.4)	2.9 (1.3)
Disease extent, *n* (%)		
Ileal	4 (14.3)	2 (13.3)
Colonic	9 (32.1)	6 (40.0)
Ileocolonic	15 (53.6)	7 (46.7)
Disease behavior, *n* (%)		
Nonstenosing, nonpenetrating	16 (57.1)	9 (60.0)
Stenosing	6 (21.4)	4 (26.7)
Penetrating	0 (0)	0 (0)
Stenosing and penetrating	6 (21.4)	2 (13.3)
History of perianal disease, *n* (%)	9 (32.1)	2 (13.3)
History of gastrointestinal surgery, *n* (%)	14 (50.0)	6 (40.0)
Prior therapy, *n* (%)		
Corticosteroids	28 (100.0)	15 (100.0)
5-ASA	16 (57.1)	10 (66.7)
Thiopurine	24 (85.7)	13 (86.7)
Methotrexate	19 (67.9)	9 (60.0)
TNF-antagonist	27 (96.4)	15 (100.0)
Vedolizumab	10 (35.7)	6 (40.0)
Natalizumab	1 (3.6)	0 (0)
Prior exposure to 0 biologics	0 (0)	0 (0)
Prior exposure to 1 biologic	2 (7.1)	1 (6.7)
Prior exposure to 2 biologics	6 (21.4)	4 (26.7)
Prior exposure to 3 biologics	15 (53.6)	9 (60.0)
Prior exposure to 4 biologics	4 (14.3)	1 (6.7)
Concomitant therapy at re-induction, *n* (%)		
Thiopurine	6 (21.4)	3 (20.0)
Methotrexate	7 (25.0)	5 (33.3)
Corticosteroid	8 (28.6)	4 (26.7)
Reason for re-induction, *n* (%)		
Active clinical symptoms and active endoscopic disease	7 (25.0)	7 (46.7)
Active clinical symptoms only	5 (17.9)	5 (33.3)
Active endoscopic disease only	3 (10.7)	1 (6.7)
Active endoscopic disease on capsule	1 (3.6)	0 (0)
Routine transition to care following clinical trial	6 (21.4)	1 (6.7)
Restarting ustekinumab following interruption in therapy	6 (21.4)	1 (6.7)

Abbreviations: 5-ASA, aminosalicylic acids; BMI, body mass index; IQR, interquartile range; TNF, tumor necrosis factor.

### Safety

We evaluated a mean 58.1 patient months of exposure to ustekinumab, including a mean 28.7 months (SD 20.3) prior to re-induction and a mean 29.4 months (SD 16.9) following re-induction.

Within 8 weeks after the ustekinumab re-induction dose, 10.7% of patients reported at least 1 AE (*n* = 5 events). AEs included 1 community-acquired pneumonia treated as an outpatient assessed as possibly related to ustekinumab, 2 traumatic injuries assessed as unlikely related to ustekinumab, 1 peristomal skin irritation assessed as unlikely related to ustekinumab, and 1 miscarriage. The miscarriage occurred in a pregnant patient of advanced maternal age who had discontinued ustekinumab for 4 months, resulting in a CD flare prior to the re-induction dose. The miscarriage was assessed as unlikely related to ustekinumab. One patient experienced SAEs; a single patient was hospitalized 3 times for CD flare and acute on chronic abdominal pain. This was assessed as unlikely related to ustekinumab.

Between 8 weeks after the re-induction dose and the end of data collection, 35.7% of patients experienced 1 or more AE (*n* = 13 events) and 7.1% experienced an SAE (*n* = 2 events). No new safety signals were identified (Table 2).

**Table 2.** Adverse events in patients with Crohn’s disease during treatment with ustekinumab, prior to and following re-induction dosing.

	Any dosing, *N* = 28	Prior to re-induction, *N* = 28	After re-induction, *N* = 28		
			Any time after re-induction	Within 8 weeks after re-induction	≥8 weeks after re-induction
Number of adverse events (AEs), *n*	130	109	18	5	13
Proportion of patients reporting AE, *n* (%)	24 (85.7)	24 (85.7)	13 (46.4)	3 (10.7)	10 (35.7)
Infection	55	49	5	1	4
Upper respiratory infection	33	30	3	0	3
Shingles	6	5	1	0	1
Gastroenteritis	3	3	0	0	0
*C. difficile* infection	3	3	0	0	0
Cellulitis	1	1	0	0	0
Sinusitis	2	2	0	0	0
Tinea pedis	2	2	0	0	0
Pneumonia	1	0	1	1	0
Tooth infection	1	1	0	0	0
Strep throat	1	1	0	0	0
Bronchitis	1	1	0	0	0
Worsening Crohn’s disease symptoms	12	12	0	0	0
Injection site pain	3	3	0	0	0
Headache	2	2	0	0	0
Rash	4	4	0	0	0
Bartholin gland abscess	1	1	0	0	0
Onychoschizia	1	1	0	0	0
Joint pain	1	1	0	0	0
Bacterial vaginosis	1	0	1	0	1
Worsened gout	1	1	0	0	0
Other AEs	49	35^a^	12	4^b^	8^c^
Number of serious adverse events (SAEs), *n*	13	8	5	3	2
Proportion of patients reporting SAE, *n* (%)	8 (28.6)	7 (30.4)	3 (10.7)	1 (3.6)	2 (7.1)
Hospitalization for worsened Crohn’s symptoms	9	6	3	3	0
Crohn’s disease flare secondary to enteropathogenic *E. coli*	1	0	1	0	1
Anaphylaxis to gadolinium	1	1	0	0	0
Adrenal insult secondary to viral infection	1	1	0	0	0
Partial small bowel obstruction	1	0	1	0	1

Traumatic injury (*n* = 6), rash (*n* = 3), joint pain (*n* = 3), traveler’s diarrhea (*n* = 2), skin cancer (*n* = 2), fatigue (*n* = 1), dizziness (*n* = 1), confusion (*n* = 1), worsened anxiety (*n* = 1), constipation (*n* = 1), preterm delivery secondary to fall (*n* = 1), liver test elevation (*n* = 1), planned ileal resection (*n* = 1), deep vein thrombosis (*n* = 1), acne (*n* = 1), worsened GERD (*n* = 1), other (*n* = 8).

Traumatic injury (*n* = 2), peristomal skin irritation (*n* = 1), miscarriage (*n* = 1).

Rectal prolapse (*n* = 1), rash (*n* = 2), tinnitus (*n* = 1), ovarian cyst (*n* = 1), impacted wisdom tooth (*n* = 1), psoriatic arthritis (*n* = 1), medial epicondylitis (*n* = 1).

For patients receiving any dosing of ustekinumab, we observed 95.7 AEs per 100 patient years. For patients receiving ustekinumab prior to the re-induction dose, we observed 163.3 AEs per 100 patient years. For patients receiving ustekinumab after the re-induction dose, we observed 26.1 AEs per 100 patient years.

### Effectiveness

Of the study population, 5 patients had both active clinical and endoscopic disease as well as adequate follow-up data to assess both clinical and endoscopic outcomes. Eight patients had active clinical disease and adequate data to assess clinical outcomes only. Two patients had active endoscopic disease and adequate follow-up data to assess endoscopic outcomes only.

**Figure 1.**
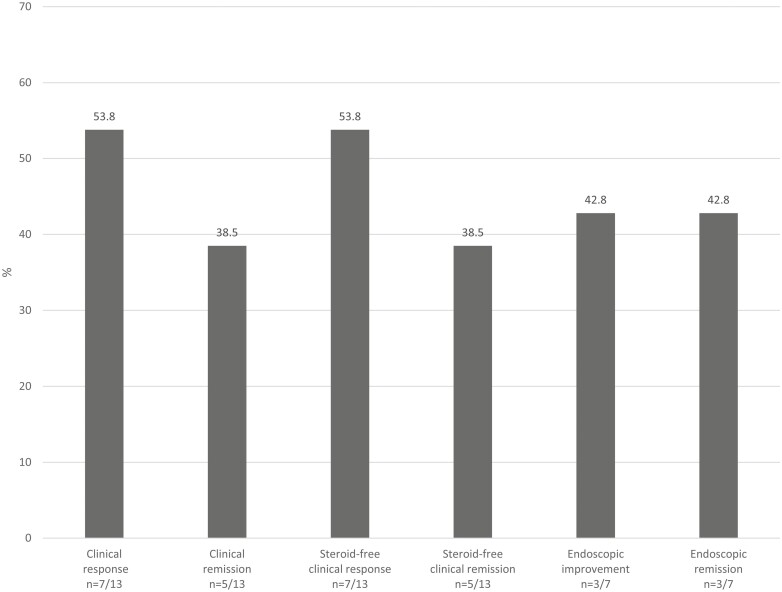
Clinical and endoscopic response after ustekinumab re-induction in patients with active Crohn’s disease prior to re-induction and adequate follow-up data to assess outcomes.

Of those with clinical data (*n* = 13), clinical response and steroid-free clinical response was achieved in 53.8% and clinical remission and steroid-free clinical remission was achieved in 38.5% of patients after a median 16.6 weeks (IQR 11.9–30.3) following re-induction. The mean HBI score improved significantly from 10.0 (SD 4.7) prior to treatment to 8.0 (SD 6.4) following re-induction (*P* = .03). Of those with endoscopic data (*n* = 7), 42.8% of patients achieved both endoscopic improvement and endoscopic remission, and none achieved endoscopic healing after a median 43.4 weeks (IQR 31.1–51.4) following re-induction. The mean SESCD score improved from 18.4 (SD 16.9) prior to treatment to 13.6 (SD 16.2) following ustekinumab re-induction (*P* = .09) (Figure 1). For patients with abnormal laboratory findings at baseline, no patients normalized CRP (*n* = 5), and 33.3%, and 33.3% patients normalized albumin (*n* = 3), and hematocrit (*n* = 6), respectively, after a median 19.6 weeks (IQR 14.7–37.1) following re-induction. The mean SIBDQ score improved from 46.5 (SD 14.5) prior to treatment to 49.4 (SD 12.7) following ustekinumab re-induction (*P* = .18).

### Pregnancy

In 3 patients, ustekinumab re-induction was administered during pregnancy at a mean 13.4 (SD 2.0) weeks of gestation. For 2 of these, re-induction was recommended to treat to CD flare in the setting of pregnancy, resulting in live birth at term without complication. In a third pregnancy, re-induction was recommended for a patient who had self-discontinued ustekinumab for 4 months, resulting in a flare of CD prior to re-induction. This pregnancy was complicated by advanced maternal age and active maternal ileocolonic and perianal CD. This pregnancy resulted in miscarriage at 12 weeks.

Six additional pregnancies occurred a mean 1.3 years (SD 1.1) after the ustekinumab re-induction dose. None of these patients received re-induction during the gestational period. The outcome was live birth at term without complication (*n* = 2), live preterm birth without complication (*n* = 1), preterm birth complicated by maternal gestational diabetes and hypertension (*n* = 1), and miscarriage (*n* = 1). One pregnancy is ongoing without complications.

### Therapeutic Drug Monitoring

Prior to re-induction, the mean ustekinumab drug level was 1.78 µg mL^−1^ (SD 1.65) and no patients had anti-ustekinumab antibodies (*n* = 11) (Table 3). No therapeutic drug monitoring was available after re-induction as this was not completed per standard clinical care.

**Table 3.** Trough ustekinumab drug levels in patients with Crohn’s disease treated with ustekinumab via sponsored clinical trial, taken at the last study visit prior to transition to clinical practice.

Patient	Ustekinumab maintenance dose (subcutaneous injection)	Ustekinumab drug level (µg mL^−1^)	Anti-ustekinumab antibodies
1	90 mg every 12 weeks	1.40126	Negative
2	90 mg every 8 weeks	0.93871	Negative
3	90 mg every 8 weeks	2.17520	Negative
4	90 mg every 8 weeks	4.87602	Negative
5	90 mg every 8 weeks	0.80600	Negative
6	90 mg every 8 weeks	4.97735	Negative
7	Did not receive maintenance dosing	0.62038	Negative
8	90 mg every 12 weeks	0.80099	Negative
9	90 mg every 12 weeks	1.71819	Negative
10	90 mg every 8 weeks	1.07211	Negative
11	90 mg every 8 weeks	Below level of detection	Negative

## Discussion

Dose optimization of ustekinumab may benefit patients with inadequate response or secondary loss of response to standard dosing, or patients with dose interruption. In our study, re-induction with ustekinumab was well tolerated. We report no new or significant safety concerns following re-induction with ustekinumab, including no infusion reactions or delayed infusion reactions in our population. While we observed AEs in pregnant patients following ustekinumab re-induction, the risk of complications in pregnant patients with active CD is well described and we did not observe any new safety concerns during pregnancy. We acknowledge that the sample size is small, making it difficult to draw definitive conclusions.

Ustekinumab re-induction improved outcomes in some patients with active disease prior to re-induction. In our study after re-induction, 53.8% of patients achieved clinical response and 38.5% achieved clinical remission. This is similar to another retrospective study of 53 patients reporting that 52.8% had clinical response and 43.3% were in clinical remission following ustekinumab re-induction.^11^ Our results are lower than another retrospective study of 15 patients reporting 78.6% of patients achieved clinical response, 57.1% achieved clinical remission, 87.5% achieved endoscopic or imaging response, and 63.5% achieved endoscopic or imaging remission following ustekinumab re-induction.^14^ Notably, in the IM-UNITI clinical trial, 46.4% of patients receiving sham adjustment of the ustekinumab maintenance dose had clinical response, compared to 55.2% of patients who received true escalation from 90 mg every 12 weeks to 90 mg every 8 weeks; 32.1% receiving sham adjustment achieved clinical remission versus 41.4% who received true dose adjustment.^2^

In our study, 42.8% achieved endoscopic improvement and endoscopic response. Notably, only those patients with active clinical or endoscopic disease at baseline were included in our clinical/endoscopic assessment. Some patients who transitioned from the blinded clinical trial did not have clinical or endoscopic disease activity prior to re-induction, but given investigators were blinded to the induction regimen and ustekinumab drug levels, we opted to treat all patients with the standard FDA-approved induction dose, assuming some patients might not have optimal drug levels. Clearly, there was variability in the therapeutic drug monitoring in our population prior to study completion, highlighting the variability in drug levels even in a standardized study protocol regimen.

While other retrospective studies have found similar symptomatic improvement with re-induction,^10–13,15^ one strength of our study is that we report the largest series of endoscopic disease activity outcomes following re-induction of which we are aware. As the primary goal of therapy in CD is endoscopic improvement rather than symptom improvement, we feel this is a critical finding. Additionally, our duration of follow-up is longer than previous studies, providing the longest longitudinal safety evaluation after re-induction of ustekinumab.

Study limitations include retrospective study design, small sample size, inherent bias in clinical decision-making, lack of comparator, and difficulty in blinding data interpretation. Future studies should consider the impact of ustekinumab maintenance dosing and therapeutic drug levels on the rate of response to re-induction. Study strengths include a long duration of safety follow-up, assessment of patient-reported symptoms, and objective evaluation of CRP and endoscopic response to therapy. Therapeutic drug monitoring was not assessed for all patients given this was not routinely complete per standard clinical practice. Notably this study shows no new safety signals, nor any dose-related safety signals, with a mean 2.5 years (SD 1.4) of follow-up after re-induction. Further randomized controlled trials are ongoing to support the efficacy and safety of ustekinumab re-induction in CD.

## Conclusion

Ustekinumab re-induction was well tolerated. No new safety signals were observed within 8 weeks of re-induction or after a mean 2.5 years of follow-up. Clinical and endoscopic disease activity improved in some patients with abnormal baseline findings. Ustekinumab re-induction may be an option for patients with inadequate response or loss of response to standard ustekinumab therapy, or in patients who need to restart therapy after dose interruption. Randomized, placebo-controlled trials are needed to assess the safety and efficacy of this treatment strategy.

## Data Availability

The data underlying this article cannot be shared publicly for the privacy of individuals that participated in the study. The data will be shared on reasonable request to the corresponding author.
